# Prevalence and characterization of antimicrobial-resistant *Escherichia coli* in chicken meat from wet markets in Hong Kong

**DOI:** 10.3389/fvets.2024.1340548

**Published:** 2024-01-15

**Authors:** F. M. Yasir Hasib, Ioannis Magouras, Sophie St-Hilaire, Surya Paudel, Maedeh Kamali, Kittitat Lugsomya, Hoi Kiu Lam, Ibrahim Elsohaby, Patrick Butaye, Omid Nekouei

**Affiliations:** ^1^Department of Infectious Diseases and Public Health, Jockey Club College of Veterinary Medicine, City University of Hong Kong, Kowloon Tong, Hong Kong SAR, China; ^2^Department of Pathology and Parasitology, Faculty of Veterinary Medicine, Chattogram Veterinary and Animal Sciences University, Chittagong, Bangladesh; ^3^Veterinary Public Health Institute, Department of Clinical Research and Veterinary Public Health, Vetsuisse Faculty, University of Bern, Bern, Switzerland; ^4^Department of Pathobiology, Pharmacology and Zoological Medicine, Faculty of Veterinary Medicine, Ghent University, Merelbeke, Belgium

**Keywords:** wet market, chicken, antimicrobial resistance, *Enterobacteriaceae*, prevalence

## Abstract

Given the close contact between animals, animal products, and consumers in wet markets, fresh meat products are considered a potential source and disseminator of antimicrobial-resistant (AMR) bacteria near the end of the food chain. This cross-sectional study was conducted to estimate the prevalence of select AMR-*E. coli* in fresh chicken meat collected from wet markets in Hong Kong and to determine target genes associated with the observed resistance phenotypes. Following a stratified random sampling design, 180 fresh half-chickens were purchased from 29 wet markets across Hong Kong in 2022 and immediately processed. After incubation, selective isolation was performed for extended-spectrum β-lactamase producing (ESBL), carbapenem-resistant (CRE), and colistin-resistant (CSR) *E. coli*. The bacterial isolates were identified using matrix-assisted laser desorption/ionization time-of-flight mass spectrometry (MALDI-TOF MS). Disc Diffusion was used to determine the susceptibility of ESBL- and CRE-*E. coli* isolates. The broth microdilution method was used to determine the minimum inhibitory concentration of CSR-*E. coli*. Targeted resistance genes were then detected by PCR. The prevalence of ESBL-*E. coli* and CSR-*E. coli* were estimated at 88.8% (95% CI: 83.4–93.1%) and 6.7% (95% CI: 3.5–11.4%), respectively. No CRE-*E. coli* isolate was detected. The *bla*_CTX-M-1_ gene was the most common β-lactamase group in isolated *E. coli* (80%), followed by *bla*_TEM_ (63.7%); no *bla*_SHV_ gene was detected. Forty-five percent of the isolates had *bla*_TEM_ and *bla*_CTX-M-1_ simultaneously. The *mcr-1* gene was detected in all 12 CSR isolates. Of 180 meat samples, 59 were from Mainland China, and 121 were locally sourced. There was no statistically significant difference in the prevalence of ESBL- and CSR-*E. coli* between the two sources. Our findings can be used to inform food safety risk assessments and set the stage for adopting targeted control and mitigation measures tailored to the local wet markets.

## Introduction

Wet markets play an important role in food culture and economy in Southeast and East Asia. Such markets are typically located in residential neighborhoods, constituting one of the main sources of fresh food (1). In Hong Kong, wet markets are regulated by the Food and Environmental Hygiene Department (FEHD) to ensure food safety and hygiene standards (2). Wet markets have recently been under increasing public scrutiny regarding food safety and hygiene concerns, especially after the outbreak of COVID-19 in Wuhan (3). Typical unsafe characteristics of some local wet markets include holding and slaughtering live animals (especially chickens and fish), mixing species, close contact between consumers and animals or their products (e.g., fresh meat), narrow corridors, poor ventilation, insufficient cooling, and wet floors (4). Given the close contact between animals, animal products and consumers in wet markets, fresh meat products are considered a potential source and disseminator of pathogens and antimicrobial-resistant bacteria near the end of the food chain.

The World Health Organization (WHO) has categorized antimicrobial resistance (AMR) among the top 10 global public health threats facing humanity with substantial economic costs (5). Antimicrobial use (AMU) in food-producing animals is a major driver of the selection and dissemination of resistant bacteria and AMR genes (6–8). Foods of animal origin represent the main route of human exposure to foodborne bacteria and their AMR genes (if any) and are, therefore, the target of national and regional monitoring and surveillance programs (9). According to the Codex Alimentarius Commission (Codex), AMR is a food safety issue, and monitoring retail foods can provide valuable insight into foodborne AMR prevalence and patterns near the end of the food chain (10).

Chicken meat makes up about 26% of the total meat consumed in Hong Kong (11). Fresh chicken meat in the wet markets is either sourced from the local chicken farms (28 active farms) or imported from Mainland China (12, 13). Despite the regulations and control measures by FEHD to ensure the safety and quality of poultry meat sold in the local wet markets, there are growing concerns regarding the high levels of contamination with multidrug-resistant (MDR) *Enterobacteriaceae* (14). Antimicrobial-resistant *Enterobacteriaceae*, especially extended-spectrum β-lactamase (ESBL)-producing *E. coli* (ESBL-*E. coli*) are the priority bacteria of concern to public health worldwide (15). They can cause difficult-to-treat lower urinary tract infections, bacteremia and gastrointestinal infections in humans, and rectal colonization with ESBL-producing *Enterobacteria* has been a growing concern in healthy individuals (16).

Food animals are major reservoirs of ESBL-*E. coli* and their products may become contaminated at any point along the food supply chain (9). All live chickens sold in the wet markets are from local farms in Hong Kong (13). The local poultry farms have access to and use antimicrobials to treat their birds which include cephalosporins, aminoglycosides, macrolides, and tetracyclines (17). The use of growth-promoting antimicrobials is not permitted in Hong Kong (17). However, recent reports have indicated inappropriate use of antibiotics in China despite improvements in evidence-based AMU over the past years (18). Poultry farms can thus act as reservoirs for the selection and spread of AMR-*Enterobacteria* to the environment and through the meat supply chain.

Regular monitoring of AMR bacteria in retail meat is necessary to ensure consumer safety, identify novel AMR hazards, and prevent their spread. Recent studies have found a high prevalence of multidrug-resistant (MDR) *E. coli* in chicken meat (48–50%) in the wet markets of Singapore and Malaysia (19, 20). In Hong Kong, 20% of human patients were infected with ESBL-*E. coli* variants (14). However, the association between poultry and human isolates and the actual level of risk posed by exposure and consumption of contaminated meat to public health remains obscure (21, 22).

The Centre for Health Protection of the local government has conducted surveillance on AMR in food-origin bacteria over the past years. The surveillance data in 2020 showed that of 597 raw meat samples tested (chicken, beef, and pork), 60% contained ESBL-*Enterobacteriaceae*, with 64% of the isolates being *E. coli* (23). Therefore, we conducted this study to estimate the prevalence of select AMR-*E. coli* (ESBL, carbapenem- and colistin-resistant *E. coli*) in fresh chicken meat collected from wet markets in Hong Kong and determined target genes associated with the observed resistance phenotypes.

## Materials and methods

### Study design and sample collection

A cross-sectional study was designed and conducted between January and March 2022 to estimate the prevalence of the contamination of fresh chicken meats with AMR-*E. coli* in wet markets across Hong Kong. Based on the list provided by FEHD, there were 97 wet markets in Hong Kong at that time, located in the three main regions (Hong Kong Island, Kowloon, and New Territories) (24). A stratified random sampling strategy was adopted to collect the required number of chicken meat samples, representing Hong Kong geographically. The wet markets were stratified into the 18 available districts in those regions, and one or two wet markets were randomly selected from each stratum using random numbers generated in Stata v17 (StataCorp LLC, College Station, TX, USA). The minimum number of samples required was estimated at 174, assuming 87% prevalence of contamination (for ESBL-*E. coli* based on previous unpublished data), 95% confidence level, and 5% precision.

**Figure 1:**
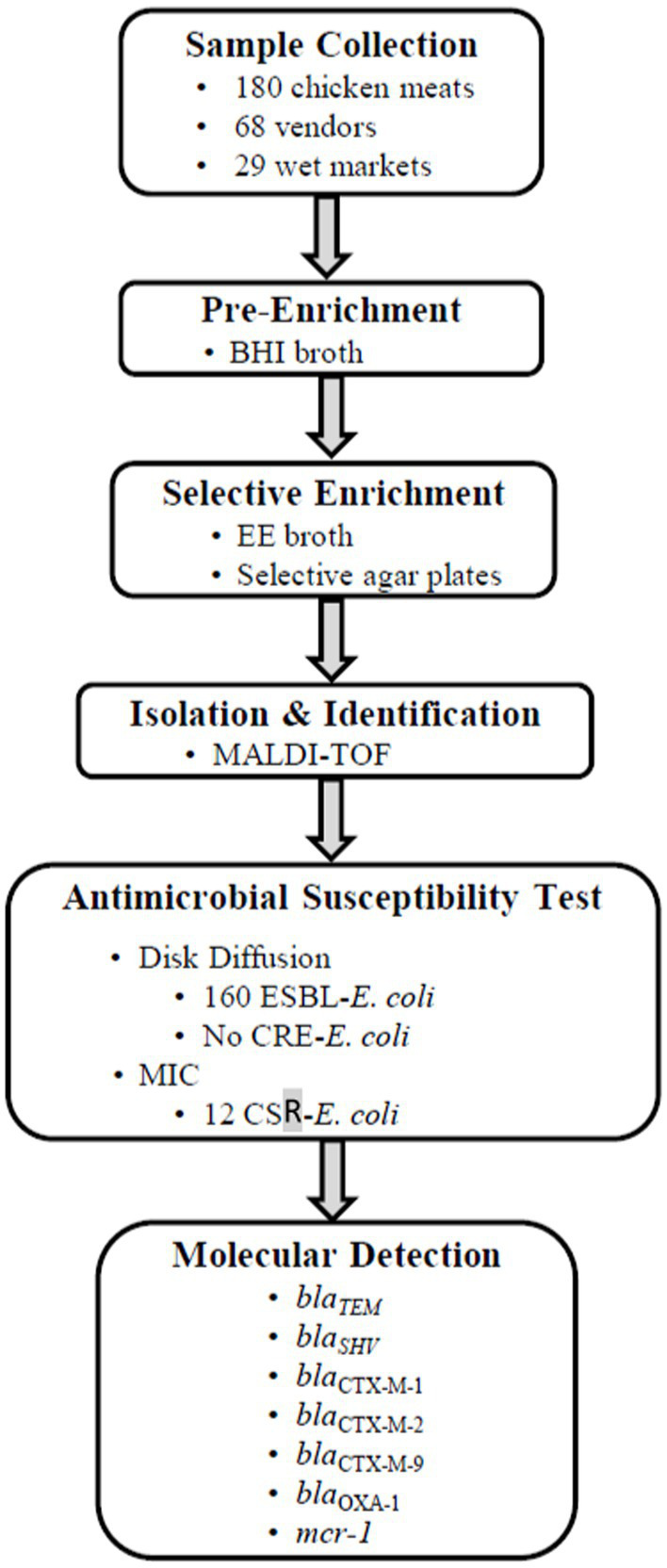
Summary of the workflow and key points in each step of the study.

In each wet market, fresh half-chickens were purchased from all available chicken meat vendors and individually packed in sterile Ziplock plastic bags. At the time of purchase, a unique ID number was assigned to each vendor, and information on the source of chicken meat (local or imported from Mainland China) was recorded. Samples were immediately delivered to the laboratory at City University of Hong Kong in an ice box. All stages of work and the corresponding key points are summarized in Figure 1.

### Sample preparation

Samples were processed within 6 h of delivery to the laboratory. Each half chicken was shredded into small pieces, with the leg, breast, and wings taken equally (50 g each), using sterile scissors, forceps, and scalpels.

### Isolation and identification of target bacteria

Three grams of shredded meat sample was mixed with 30 mL brain heart infusion (BHI) broth (Thermo Fisher Scientific, Waltham, USA) and incubated at 37°C overnight; 0.1 mL of this suspension was inoculated in *Enterobacteriaceae* Enrichment (EE) broth (Thermo Fisher Scientific, Waltham, USA) mixed with antibiotic supplements listed in Supplementary Table S1. Cultures were subsequently plated on the corresponding selective agar plates. For selective isolation of ESBL, Carbapenemases-producing (CRE) and Colistin-resistant (CSR) *E. coli*, Brilliance™ ESBL agar, Oxoid Brilliance™ CRE agar, and CHROMID® Colistin R were used, respectively (Table 1), and incubated at 37°C for 18–24 h. One typical colony was purified, and the isolate was identified by matrix-assisted laser desorption/ionization time-of-flight mass spectrometry (MALDI-TOF MS), using the MALDI Biotyper® (Bruker, MA, USA). Cultures were then stored at −80°C for further analysis.

**Table 1:** Selective broth and agar used for the isolation of antimicrobial-resistant *E. coli* and their colony characteristics.

Resistance type	*Enterobacteriaceae* Enrichment broth with antibiotic (Selective Enrichment I)	Selective agar (Selective Enrichment II)
Extended-spectrum beta-lactamases producing (ESBL)	Cefotaxime (CTX) 3.5 μg/mL	Brilliance™ ESBL
Carbapenemases producing (CRE)	Meropenem (MEM) 3.5 μg/mL	Oxoid Brilliance™ CRE
Colistin-resistant (CSR)	Colistin (CSR) 3.5 μg/mL	CHROMID® Colistin R

### Antimicrobial susceptibility test (AST)

The disc diffusion method was used to determine the susceptibility of ESBL-*E. coli* and CRE-*E. coli*, based on the European Committee on Antimicrobial Susceptibility Testing (EUCAST) guidelines. For ESBL-*E. coli*, cefotaxime (30 μg), cefotaxime-clavulanic acid (30 μg-10 μg), ceftazidime (30 μg) and ceftazidime-clavulanic acid (30 μg-10 μg) were used. For CRE- *E. coli*, meropenem (10 μg), imipenem (10 μg), ertapenem (10 μg), and doripenem (10 μg) were used. The *E. coli* ATCC® 25922 isolate was used as the reference for routine quality control of the phenotypic AST. As for CSR-*E. coli*, the minimum inhibitory concentration (MIC) was determined using the broth microdilution method as recommended by EUCAST with the breakpoint of >2 μg/mL (25).

### Plasmid extraction and molecular characterization of resistant isolates

A single colony of phenotypically resistant *E. coli* grown on Tryptic Soy Agar plates (Sigma Aldrich®, USA) was inoculated in 4 mL of BHI broth and incubated for 12–14 h at 37°C in a shaking incubator (225 RPM/min). Four ml of the overnight grown culture with an optical density (OD) value of 2.4–2.5 was used for plasmid extraction by Takara MiniBEST plasmid purification kit version 4.0 following the manufacturer’s protocol (Takara Inc., Shiga, Japan). The concentration of extracted plasmid was determined in a Nanodrop (Thermo Fisher, Waltham, USA).

Polymerase chain reaction (PCR) was done to detect *bla*_CTX-M-1_, *bla*_CTX-M-2,_ *bla*_CTX-M-9_ gene groups, as well as *bla*_OXA-1_, *bla*_SHV_, *bla*_TEM_, *mcr-1* genes. Primers and PCR conditions are presented in Table 2. Reactions were performed in a total volume of 25 μL with 12.5 μL Premix Ex Taq Hot Start Version master mix (Takara Inc., Shiga, Japan), 1 μL each of forward and reverse primer (10 μM), 1 μL plasmid DNA template and 9.5 μL of nuclease-free water (NFW), using the ProFlex™ PCR System (Thermo Fisher Scientific, MA, USA). Amplified PCR products were stained with 6X purple gel loading dye (New England Biolab Inc., MA, USA) and visualized after gel electrophoresis on 1.5% ultrapure agarose (Thermo Fisher Scientific, MA, USA).

**Table 2:** Primer sequences, expected band size, and PCR methods used for the detection of target resistance genes.

Target gene	Primer sequences (5′-3′)	Size (bp)	Condition	Reference
*bla*_TEM_	F: CATTTTCGTGTCGCCCTTATTC	800	30 cycles of 94°C for 30s; 58°C for 30s; 72°C for 1 min	(26)
	R: CGTTCATCCATAGTTGCCTGAC			
*bla*_SHV_	F: AGCCGCTTGAGCAAATTAAAC	713		
	R: ATCCCGCAGATAAATCACCAC			
*bla*_CTX-M-1_	F: TTAGGAARTGTGCCGCTGYA	688	30 cycles of 94°C for 30s; 60°C for 30s; 72°C for 1 min	(26)
	R: CGATATCGTTGGTGGTRCCAT			
*bla*_CTX-M-2_	F: CGTTAACGGCACGATGAC	404		
	R: CGATATCGTTGGTGGTRCCAT			
*bla*_CTX-M-9_	F: TCAAGCCTGCCGATCTGGT	561		
	R: TGATTCTCGCCGCTGAAG			
*bla*_OXA-1_	F: ATATCTCTACTGTTGCATCTCC	619	35 cycles of 94°C for 30s, 52°C for the 30s, 72°C for 1 min	(27)
	R: AAACCCTTCAAACCATCC			
*mcr-1*	F: AGTCCGTTTGTTCTTGTGGC	320	35 cycles of 94°C for 30 s, 58°C for 90s and 72°C for 1 min	(28)
	R: AGATCCTTGGTCTCGGCTTG			

### Statistical analysis

All data management and analyses were performed using Stata v17 (Stata Corp LLC, College Station, TX, USA). The proportion of each AMR pattern and target gene detected from the samples was defined as “prevalence.” The prevalence of each resistance pattern and gene of interest were estimated and compared between the two sources of chicken meat (local vs. Mainland China) using the tests of proportions with the significance level set at 0.05.

## Results

In total, 180 chicken meat samples were collected from 29 wet markets with 68 different vendors (Figure 1). As planned, we selected one or two wet markets per district proportional to the numbers available on the list. The number of vendors sampled per wet market ranged between one and six, with most wet markets having one or two vendors (21/29).

### Prevalence of ESBL, CRE, and CSR *Escherichia coli*

The prevalence of ESBL-*E. coli* and CSR-*E. coli* were estimated at 88.8% (95% CI: 83.4–93.1%) and 6.7% (95% CI: 3.5–11.4%), respectively. No CRE-*E. coli* was detected in the meat samples (Figure 1).

### Molecular detection of target genes

The *bla*_CTX-M-1_ gene group was the most common β-lactamase in the 160 selectively isolated *E. coli* (80%), followed by *bla*_TEM_ (63.7%), *bla*_CTX-M-9_ (22.5%), *bla*_OXA-1_ (20%), and *bla*_CTX-M-2_ (2.5%); *bla*_SHV_ was not detected. Forty-five percent of the isolates had the two β-lactamases TEM (*bla*_TEM_) and CTX-M-1, and only one strain contained four different β-lactamases simultaneously (Supplementary Figure S1). The *mcr-1* gene was detected in all 12 CSR-*E. coli* isolates.

### Source comparisons

Of the 180 meat samples, 59 were from Mainland China and 121 were locally sourced. The frequency of detected target genes by source is summarized in Table 3. There was no statistically significant difference in the prevalence of ESBL-*E. coli* and CSR-*E. coli* between the two sources (Table 3). Among the target genes detected in our study, *bla*_TEM_ and *bla*_OXA1_ had a significantly higher prevalence in samples from Mainland China compared to the locally-sourced samples, with *p* = 0.035 and *p* < 0.001, respectively. There was no statistically significant difference in the prevalence of the other target genes between the two sources of chicken meat samples (Table 3).

**Table 3:** Frequency of ESBL- and CSR-*E. coli* isolates and associated target genes from 180 chicken meat samples by source (121 from Hong Kong and 59 from Mainland China).

	Total	Hong Kong (%)^1^	Mainland China (%)^2^	*p*-value
ESBL-*E. coli*	160	109 (90.1)	51 (86.4)	0.465
*bla*_CTX-M-1_	128	83 (68.6)	45 (76.3)	0.286
*bla*_TEM_	102	62 (51.2)	40 (67.8)	0.035
*bla*_CTX-M-9_	36	26 (21.5)	10 (16.9)	0.475
*bla*_OXA-1_	32	13 (10.7)	19 (32.2)	<0.001
*bla*_CTX-M-2_	4	4 (3.3)	0 (0)	0.158
*bla*_SHV_	0	0 (0)	0 (0)	-
CSR-*E. coli* (*mcr-1*)	12	6 (4.9)	6 (10.1)	0.188

^1^ % in the parentheses represents the number of each detected gene divided by 121.

^2^ % in the parentheses represents the number of each detected gene divided by 59.

## Discussion

We investigated the contamination of fresh chicken meat sold in wet markets of Hong Kong with a number of priority AMR patterns of concern in *E. coli* as indicator bacteria. In Hong Kong, antibiotics such as amoxicillin, ampicillin, and cefquinone are used for treatment purposes in poultry production (17). While the precise amount of antimicrobial use on poultry farms in Hong Kong is unclear, penicillins and cephalosporins comprised 25.4% (36.47 kg) and 0.84% (1.2 kg) of the total antibiotic use on the farms in 2018 and 2019, according to a governmental report (17). This selection pressure may explain a part of the very high prevalence of ESBL-*E. coli* in fresh chicken meat observed in our study. Historical surveillance data also indicated similar contamination levels in Hong Kong over the past decade (77–93%) in meat products collected from various retail markets (17). This constantly high prevalence of ESBL-*E. coli* contamination in chicken meat from the markets can be indicative of shortfalls in enforcing and monitoring current hygienic and safety measures.

Detecting *E. coli* in meat products indicates direct and/or indirect fecal contamination at some point in the food chain. At the beginning of the chain, the prevalence of ESBL-*E. coli* in cloacal swabs and fecal samples from chicken farms in Hong Kong was estimated at about 25% in 2021 (17). Some wet markets sell live chickens, in which vendors are responsible for slaughtering and cutting live chickens. These vendors keep the fresh meat in a cooler or at ambient temperature to sell. Therefore, contamination may occur during the slaughtering process within the shops, particularly during the de-feathering and evisceration, where intestinal contents may come into contact with the meat and other surfaces. We also observed that vendors often use the same tools (e.g., knives, bone cutters, wooden surfaces, gloves, and feather plucking machines) during meat preparation without regular de-contamination, which could readily lead to cross-contamination. While cross-contamination is a major source of bias in estimating food contamination levels in many market-based studies, and it could have overestimated the prevalence in our study, there is no straightforward way to estimate the actual impact of potential cross-contamination sources. In market-based studies, determining contamination in salable meat, regardless of the source of contamination, is the main objective in most surveys (including ours) and surveillance programs because that contamination level is the one to be translated into the risk of exposure for the consumers. Moreover, several other factors can potentially contribute to the high levels of meat contamination in the wet markets, including overcrowding, poor air conditioning, uneven air flow, high humidity, and limited storage capacity, which should be studied further.

The low level of contamination of fresh chicken meat with colistin-resistant *E. coli* in our study (6.6.%) was consistent with the historical records (14). Prevalence of CSR-*E. coli* in chicken meat was estimated between 5 and 30% from 2011 to 2014 in China (29). After discovering a plasmid-mediated colistin resistance gene in 2015, using colistin as a growth promoter in animals was banned (30). The prevalence of CSR-*E. coli* in cloacal swabs from chickens was estimated at about 12% in 2017 and below 1% in 2019 across China, indicating a decreasing trend (31, 32). However, we should note the differences in study design and sampling strategies used in these studies that could have affected the precision of these estimates.

We did not detect any CRE-*E. coli* in our study. There is limited literature on the detection and prevalence of CRE-*E. coli* in chicken meat. The prevalence of CRE-*E. coli* from retail chicken meat in Egypt and the UK were 11.3 and 0%, respectively (33, 34). A recent study found CRE-*E. coli* in 4% of cloacal swabs obtained from broiler chickens in the Shandong Province of China (35). Since carbapenem antibiotics are not used in poultry farming, we hypothesize that these isolates could be from cross-contamination. However, it is noteworthy that β-lactam antibiotics, such as penicillin and amoxicillin, may also exert selection pressure for resistance against carbapenem (36).

In this study, we chose specific target genes based on a literature review around the most important and/or commonly detected plasmid-mediated genes linked with ESBL and CSR resistance of *E. coli* in poultry and public health to verify the observed phenotypic resistance patterns. It has been shown that the family of *bla*_CTX-M_ genes are the most prevalent in both human- and animal-origin cephalosporin-resistant bacterial isolates (37), of which *bla*_CTX-M-1_ and *bla*_CTX-M-15_ are the most common in human and poultry isolates (38, 39). The *bla*_CTX-M-2_ and *bla*_CTX-M-9_ genes were the second and third most prevalent in *E. coli* isolated from chicken meat samples (40). We also specifically targeted *bla*_OXA-1_ in our study because a community-based survey in Hong Kong detected this gene in three out of 113 *E. coli* isolates from seemingly healthy people (16). Variability in the prevalence of different *bla*_CTX-M_ genes by geographical region and animal species has been described (41). Our study showed that among targeted *bla*_CTX-M_ gene groups, *bla*_CTX-M-1_ was more prevalent compared to *bla*_CTX-M-2_ and *bla*_CTX-M-9,_ which aligns with the geographical and molecular epidemiological data from other studies (42–44). The *bla*_CTX-M-1_ gene was also the most prevalent in cloacal samples collected from chickens in Hong Kong (45). Worldwide, *bla*_CTX-M-1_ and *bla*_CTX-M-9_ groups are among the most common ESBL genes in human cases infected with *Enterobacteriaceae* in hospitals, and a decreasing trend in the detection of *bla*_CTX-M-2_ has been reported (42). While some recent studies have shown difficulty in finding the animal/food origins of these ESBL genes in *E. coli* isolated from humans, a direct or indirect transmission could not be excluded (46–48). The co-occurrence of *bla*_CTX-M-1_ and *bla*_TEM_ was very common in our ESBL-*E. coli* isolates. This co-occurrence has been frequently noted in broiler chicken samples from Southeast Asia (49, 50) and was also found in *K. pneumoniae* isolates from chickens and their environmental samples in Egypt (51). The co-occurrence of CTX-M and TEM, class A β-lactamases, may result in an extended β-lactam spectrum (52).

We found no significant differences in the prevalence of the *bla*_CTX-M_ genes between the two sources of fresh chicken meat (Mainland vs. local). However, *bla*_TEM_ and *bla*_OXA-1_ were relatively more common in Mainland-sourced chicken meats. While there is no obvious reason for these differences, some differences in genetic linkage and co-selection might exist. The emergence of OXA β-lactamases has been linked to the extensive use of flucloxacillin and methicillin in human bacteria (53). The co-presence of *bla*_OXA-1_ and *bla*_CTX-M-1_ may cause non-carbapenemases producing carbapenem resistance among the ESBL isolates; however, this was not observed in our study (54).

For the CSR-*E. coli*, *mcr-1* is the most common plasmid-mediated colistin resistance gene identified along the food chain (55). All 12 CSR-*E. coli* isolates in our study carried *mcr-1* gene on their plasmids, in agreement with other studies (56, 57). We observed that the prevalence of CSR-*E. coli* was not significantly different between mainland and local chicken meats, which may indicate a similar selection pressure in the two locations. A study conducted in Vietnamese wet markets reported a higher prevalence of CSR-*E. coli* (6/15) in chicken meat, though the very small sample size in this study should be noted (58).

Formal microbial risk assessments are required to link the contamination levels of meat with AMR bacteria with its potential public health risks (10). While it remains unclear whether *E. coli* has some host specificity, the importance of this resistance for human health may lie in the horizontal transfer of the corresponding genes (59, 60). However, there are contradictory findings from studies aiming to determine the potential transfer of AMR genes from animal-origin bacteria to human isolates. Some studies have shown possible transfers based on molecular epidemiological data, while others failed to demonstrate such transfer in specific environments (59, 61).

Although we did our best to conduct the random sampling as planned, selection bias could have affected our prevalence estimates due to the field condition. At the time of purchase, some of the randomly selected wet markets had only one vendor open (COVID era), and some others had multiple vendors selling chicken meat. We had to adapt and adjust for the number of samples required in the field. Therefore, in some wet market, more than two (up to six) vendors were sampled. This could have caused a slight clustering effect (as either under- or over-estimation) that we could not account for in our estimations, but we believe this would not have a considerable impact on the overall estimate of prevalence because only one or two vendors were sampled from the majority of selected markets in the end (21/29). In addition, the potential effect of season on the prevalence of contamination with ESBL-*E. coli* can be discussed as we conducted our study in the winter. One may argue that the level of contamination can be expected to be higher in summer. With respect to the already very high level of contamination in winter (88%), and available data from historical studies in different seasons, this issue should not be of concern in relation to our final conclusions and practical recommendations.

In conclusion, the high prevalence of ESBL-*E. coli* in fresh chicken meat sold in the wet markets in Hong Kong, and the observed lack of compliance with current hygiene and food safety regulations among many vendors urge action. Implementing antimicrobial stewardship programs may reduce the prevalence of these resistant bacteria in poultry and their products. For this, sustained monitoring of the use of antimicrobials, as well as a proper AMR surveillance program, are needed. Establishing a centralized slaughterhouse for local chickens and enforcing HACCAP on meat handling and processing practices in wet markets are recommended to mitigate the potential risks to public health. However, the actual level of risk from exposure to ESBL-*E.coli* and other AMR hazards in fresh meat must be further evaluated by formal food microbial risk analyses.

## Data availability statement

The original contributions presented in the study are included in the article/Supplementary material, further inquiries can be directed to the corresponding author.

## Ethics statement

Ethical review and approval was not required for the study in animals in accordance with the local legislation and institutional requirements.

## Author contributions

FH: Data curation, Investigation, Methodology, Writing – original draft, Writing – review & editing. IM: Conceptualization, Investigation, Methodology, Project administration, Resources, Supervision, Writing – review & editing. SS-H: Methodology, Supervision, Writing – review & editing. SP: Methodology, Supervision, Writing – review & editing. MK: Investigation, Methodology, Writing – review & editing. KL: Investigation, Methodology, Writing – review & editing. HL: Data curation, Investigation, Methodology, Writing – review & editing. IE: Methodology, Writing – review & editing. PB: Methodology, Validation, Writing – review & editing. ON: Conceptualization, Data curation, Formal analysis, Investigation, Methodology, Project administration, Resources, Software, Supervision, Visualization, Writing – original draft, Writing – review & editing.
