# Supplementary figures and images for: Prevalence and characterization of antimicrobial-resistant Escherichia coli in chicken meat from wet markets in Hong Kong
Source: Front Vet Sci. 2024 Jan 15;11:1340548. doi: 10.3389/fvets.2024.1340548 (PMC10822974; doi:10.3389/fvets.2024.1340548)

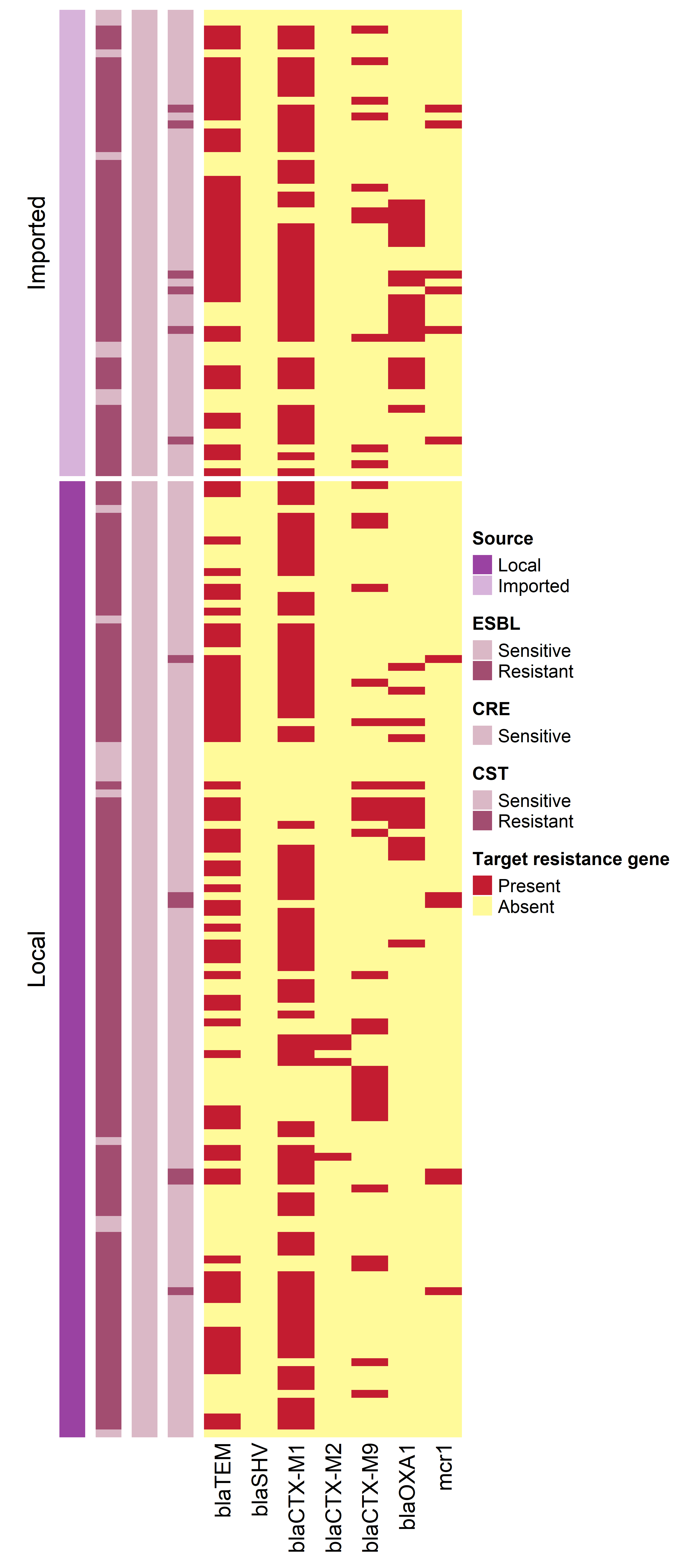

Supplement: Supplementary file 2 [file Image_1.TIF]
